# Sensory approaches in psychiatric units: Patterns and influences of use in one Australian health region

**DOI:** 10.1111/1440-1630.12813

**Published:** 2022-06-15

**Authors:** Lisa Wright, Pamela Meredith, Sally Bennett

**Affiliations:** ^1^ School of Health and Rehabilitation Sciences The University of Queensland Brisbane Queensland Australia; ^2^ The Prince Charles Hospital Metro North Mental Health Hospital and Health Services Brisbane Queensland Australia; ^3^ School of Health and Behavioural Sciences University of Sunshine Coast Sunshine Coast Queensland Australia

**Keywords:** inpatient care, mental health, patterns of use, sensory approaches

## Abstract

**Background/aim:**

Australian guidelines and policies recommend the use of sensory approaches in mental health care. Nevertheless, many Australian psychiatric units report difficulty sustaining these approaches. To inform efforts to close the gap between recommendations and practice, the aim of this study was to understand the patterns of use of sensory approaches and what demographic and clinical factors influence their use, across one health region in Queensland, Australia.

**Methods:**

Using a cross‐sectional survey design, a custom‐designed questionnaire was distributed via email and in paper form to health professionals and peer support workers working in acute, secure, and community care psychiatric units across one health region. Information on demographics and the use of various sensory interventions was gathered utilising both open‐ended and Likert scale questions.

**Results:**

Useable questionnaires were collected from 183 participants from various disciplines (77% nursing). The majority reported using sensory approaches with a limited number of consumers, and almost 9% *never* used the approach. Activity‐based sensory interventions and sensory equipment were most often used, whereas sensory assessments, sensory plans, and sensory groups were least used. Sensory interventions were mainly used to reduce consumer anxiety and agitation and to assist with emotional regulation. Factors positively correlated with frequency of use for all interventions were discipline (occupational therapy); working in an acute inpatient unit; and training in sensory approaches. Age was negatively correlated with frequency of use only for weighted modalities.

**Conclusions:**

This study revealed that sensory approaches were used by most staff though with a limited proportion of consumers in psychiatric units in one large metropolitan health service. It provides insights into the factors influencing frequency of use, highlighting the importance of training in sensory approaches and access to occupational therapists. With this knowledge, we can work towards closing the gap between recommendations and the practice of sensory approaches.

**Key points for occupational therapy**

- Most health professionals provided sensory interventions to <25% of consumers with whom they worked.
- Occupational therapists are driving implementation of sensory approaches in mental health care.
- Occupational therapists should consider providing initial and refresher training to their colleagues to improve use of sensory approaches.

## 1 INTRODUCTION

National and state guidelines, frameworks, standards, and policies support the use of sensory approaches in inpatient psychiatric units across Australia (Australian Health Ministers' Advisory Council, 2016; New South Wales Government, 2020; Queensland Health, 2008, 2018; South Australian Health, 2017; Tasmanian Health, 2013; Victorian Health, 2017; Western Australian Health, 2015). The recommendations in government documents to use sensory approaches have been fuelled by the national agenda to reduce and, if possible, eliminate the use of restrictive practices (such as seclusion and restraint) in Australian psychiatric units (Australian Health Ministers' Advisory Council, 2016). The use of restrictive practices can cause significant harm to an individual and seriously breaches their human rights (Maker & Mcsherry, 2019). The United Nations has called for a total ban on the use of restrictive practices (Méndez & Mendez, 2013), so it is imperative to implement evidence‐based alternatives to the practice, such as sensory approaches. The aim of sensory approaches is to support recovery through self‐management of distress and arousal, improve a person's mental state, and assist with the reduction and/or elimination of restrictive practices (Australian Commission on Safety and Quality in Health Care, 2017; Australian Health Ministers' Advisory Council, 2016; Australian Health Minister's Advisory Council, 2013). Sensory approaches recommended for use in psychiatric units include sensory rooms, sensory carts, and safety plans that identify individualised sensory strategies. Evidence suggests that such sensory approaches are safe, effective, and valuable interventions for mental health consumers (Craswell et al., 2020). Despite emerging empirical support for the use of sensory approaches, and the recommendations for their use in government policies and guidelines, implementation and sustainability of sensory approaches in mental health settings have faced challenges (Australian College of Mental Health Nurses, 2017; Wright et al., 2017, 2020). The aim of this study is to investigate how sensory approaches are used in clinical practice in psychiatric units and explore clinical and demographic factors that influence their use. This will inform efforts to close the gap between recommendations and practice in inpatient psychiatric units to support care of mental health consumers.

‘Sensory approaches’ is an umbrella term that covers a broad range of sensory interventions. *Sensory interventions* are those specific clinical techniques and strategies that are: based on an understanding of sensory systems; recovery‐focussed; person‐centred; and trauma‐informed (Champagne, 2011). They involve working collaboratively with consumers to identify their unique sensory preferences and develop individualised sensory strategies to support self‐management (Champagne, 2011). Sensory interventions typically focus on using the environment, sensory tools or equipment, and sensory‐based activities to improve emotional regulation and reduce distress (Sutton & Nicholson, 2011). Accumulating evidence, including two recent randomised controlled trials (RCTs), supports the use of sensory approaches in mental health care. In the first RCT, Cheng et al. (2017) demonstrated the therapeutic benefits of sensory rooms in reducing anxiety and negative symptoms of schizophrenia. The second RCT (Bensimon et al., 2018) demonstrated the therapeutic use of music in seclusion rooms in reducing consumer psychomotor agitation and promoting a sense of calm. Evidence further suggests that using sensory interventions can reduce distress and agitation (Cummings et al., 2010), decrease arousal levels (Dorn et al., 2020), promote a sense of calm (Hedlund Lindberg et al., 2019), and contribute to reduced use of seclusion and restraint in psychiatric units (Lloyd et al., 2014). Despite evidence for the effectiveness of sensory interventions, some Australian psychiatric units have been unable to sustain their use (Wright et al., 2017, 2020). In a New South Wales Government report by Wright et al. (2017), sensory rooms were found to be poorly maintained and underutilised. Similarly, Wright et al. (2020) found that some sensory rooms and sensory kits were no longer used due to poor maintenance and loss of sensory equipment.

In Australia, national principles for the elimination of restrictive practices recommend the use of sensory approaches to manage distress and/or arousal in mental health settings, including the use of personal safety plans which identify warning signs, triggers, and individualised sensory strategies (Australian Health Ministers' Advisory Council, 2016). These principles also recommend that mental health clinicians be trained in the use of sensory approaches. In addition, many of the states of Australia have developed Chief Psychiatrist policies/standards/guidelines detailing the legal use of restrictive practices (such as seclusion and mechanical and physical restraint) (Mental Health Act (Qld), 2016; South Australian Health, 2017; Tasmanian Health, 2013; Victorian Health, 2017; Western Australian Health, 2015). These documents identify sensory interventions (such as sensory rooms/carts/trolleys and personal safety plans) as one strategy to reduce and eliminate the use of seclusion and restraint in psychiatric units.

The aim of government mental health care policy, guidelines, and standards is to improve the quality of health care and ensure clinical interventions are informed by the best available evidence (World Health Organization, 2007). Implementation of evidence‐based care and government policy recommendations is known to be challenging across the health‐care sector (Grol & Grimshaw, 2003), and mental health services are a prime example (Sandström et al., 2015). For example, some mental health services have experienced difficulty implementing evidence‐based practices such as recovery‐orientated practice and the Safewards model (Higgins et al., 2018; Slade et al., 2014).

The use of implementation science theories and frameworks can assist the investigation of challenges to implementation in health care (Nilsen, 2015). According to implementation science, one of the first steps to solving implementation problems is to understand the knowledge‐practice gap, that is, the gap between the evidence and actual practice (Graham et al., 2006). Once a knowledge‐practice gap is established, further investigation is required to ascertain the factors influencing implementation of the desired practice (Graham et al., 2006). To understand factors influencing the use of sensory approaches, a qualitative study was conducted with 15 Australian mental health professionals working in inpatient psychiatric units (Wright et al., 2020). This study revealed four salient factors, listed here in order of importance: social influences (*e.g., having support from peers to use sensory approaches*), belief about consequences (*e.g., belief that sensory approaches are effective*), professional role and identity (*e.g., beliefs about whose role it is to provide sensory approaches*), and the environmental context and resources (*e.g., availability of sensory equipment*) (Wright et al., 2020). In addition, this research highlighted within‐service variation in implementation of sensory approaches, with some units having established sensory interventions as routine practice while others struggled to sustain their use.

Little is known about the pattern of use of sensory interventions (*i.e., what proportion of patients it is used with, types of approaches used, frequency and purpose of their use*) in routine clinical practice in Australian psychiatric units. Indeed, no studies could be found *internationally* that investigated the pattern of use of sensory interventions in psychiatric units. The aim of the present study was to understand the patterns of use of sensory approaches and what demographic and clinical factors influence their use, in one mental health region. Knowledge of these factors may inform development of strategies to improve the use and sustainability of sensory approaches in mental health inpatient units and, ultimately, decrease the use of seclusion and restraint.

The specific research questions, relating to one health region in Australia, were:

1. With what proportion of consumers in psychiatric units do mental health clinicians use sensory interventions?
2. What types of sensory interventions are used by these mental health clinicians?
3. How frequently, and for what purpose, are different types of sensory interventions used?
4. What demographic and clinical factors (unit type, discipline, gender, level of training, age, and years of experience in mental health) are associated with these patterns (type and frequency) of use?

## 2 METHODS

### 2.1 Design

This paper is one of two emerging from a cross‐sectional survey of mental health clinicians and peer support staff working in acute and rehabilitation psychiatric units across one health region in Queensland. The other paper addresses barriers and enablers to the use of sensory approaches (Wright et al., 2021). Ethical clearance was obtained from The Prince Charles Hospital, Queensland Health (HREC/17/QPCH/297) and The University of Queensland (#2017001711), Queensland, Australia.

### 2.2 Data collection

#### 2.2.1 Participants

Participants were a convenience sample recruited between July and September 2019 from three major psychiatric hospitals, including staff working in acute inpatient units, acute care teams, secure mental health rehabilitation units, and community care rehabilitation units, in Queensland, Australia. As the focus of the research was on the psychiatric inpatient units, alcohol and drug staff and continuing care mental health staff were excluded unless they also worked within an acute service.

#### 2.2.2 Instrument

Following a review of the literature, including government guidelines/policies, on sensory approaches in mental health care, a questionnaire was developed, which could be completed online or in paper form. The full questionnaire contained six sections, of which four are considered in this paper. Section 1 contained just one question that asked about the proportion of their patients (from 0% to over 75%, see Table 1) with whom participants used sensory interventions. In Section 2, participants were asked about the types of sensory interventions they used using one open‐ended and one forced‐choice question (i.e., non‐weighted sensory interventions, weighted modalities, sensory room, sensory groups, and sensory assessments). Participants could also indicate if these options were unavailable in their unit. Section 3 consisted of 15 questions asking about *frequency* of use of sensory interventions, and frequency of use of sensory interventions for specific reasons (e.g., to reduce anxiety and agitation), using a 5‐point Likert scale (1 = *Never* to 5 = *Very frequently*). These 15 questions were divided into subscales regarding use of (1) sensory interventions overall (5 questions), (2) weighted modalities (3 questions), (3) sensory assessments (5 questions), (4) sensory room (1 question), and (5) sensory groups (1 question). The fourth section used in this study, Section 6, sought demographic information (age, gender) and occupational (work area, position level, years of experience in mental health care, and the amount of training they had completed about sensory approaches). Participants indicated which courses, if any, they had attended (including *internal*: full‐day Sensory Awareness Workshop, half‐day new graduate training, ward‐based in‐services, and weighted modalities competency in‐services; and *external*: training in sensory approaches).

**TABLE 1:** Participants' characteristics, *n* = 183

Variable	Category/range	*n*	%	Mean (SD)
Gender	Female	124	67.8	
	Male	49	26.8	
	Other	1	0.5	
	Missing	9	4.9	
Age	Age (range: 21–67 years)	165	90.1	39.0 years (12.89)
	Missing	18	9.8	
Years working in mental health (experience)	Experience (range: 4 weeks–40 years)	165	90.1	8.61 years (9.12)
	Missing	18	9.8	
Discipline	Nursing	141	77.0	
	Occupational therapy	12	6.6	
	Psychology	8	4.4	
	Social work	7	3.8	
	Peer support workers	6	2.2	
	Other	5	2.7	
	Medical	4	2.2	
	Missing	1	0.5	
Area of work	Acute inpatient unit	125	68.2	
	Community care unit	26	14.2	
	Secure mental health unit	15	8.2	
	Community	6	3.3	
	Acute care team	7	3.8	
	Integrated (work across both hospital and community)	4	2.2	
Hospital	Hospital 1	77	42.1	
	Hospital 2	57	31.1	
	Hospital 3	49	26.8	
Attended training	Trained (attended at least one session)	111	60.7	
	Never	63	34.4	
	Missing	9	4.9	
Amount of training	One session only (range: 1–6 sessions)	71	38.8	1.04 (1.17)
	Two sessions	24	13.1	
	Three sessions	9	4.9	
	Four to six sessions	7	3.8	
	Missing	9	4.9	
Sensory training attended	Introductory sensory awareness 1 day	34	18.6	
	Half day graduate training	31	16.9	
	Ward/team‐based in‐services	28	15.3	
	QCMHL^a^ online module	22	12.0	
	Other training (sensory workshop attended elsewhere, one on one training, conference presentations)	19	10.4	
	Weighted modalities competency training	18	9.8	
	Tina Champagne sensory training	7	3.8	
	Pearson's Sensory Profile training^b^	2	1.0	
	Missing	9	0.2	
Proportion of consumers sensory interventions used with	0%	16	8.7	
	1–10%	36	19	
	11–25%	33	18	
	26–50%	33	18	
	50–75%	28	17.2	
	Over 75%	17	10.4	
	Missing	20	10.9	

^a^ QCMHL Queensland Centre for Mental Health Learning.

^b^ Pearson's Sensory Profile training is only attended by occupational therapists and psychologists.

#### 2.2.3 Procedures

The questionnaire was distributed electronically (via Consultation Hub) and in paper form at staff meetings to clinical staff and peer workers working in inpatient psychiatric units across three hospital sites within one mental health service district. Completed paper questionnaires were returned to the first author anonymously in a sealed survey box made available at the various clinical sites. Potential participants were provided with a Participant Information Statement before commencing the questionnaire indicating participation was voluntary, with completion and return of the questionnaire indicating consent.

### 2.3 Data analysis

IBM SPSS for Windows version 25 (IBM Corp, Released, 2017) was used for quantitative analyses. For descriptive purposes, frequency responses were collapsed from 5‐point Likert scales to 3‐point scales yielding a scale of 1 = *never*; 2 = *rarely/sometimes*; and 3 = *frequently/very frequently*. The amount of training attended was calculated as the number of different training sessions (regardless of length) completed. Confirmatory factor analysis was performed on the three frequency subscales with more than one item (i.e., *sensory interventions overall*, *weighted modalities*, and *sensory assessments*) to confirm relationships between variables in each subscale, and internal consistency (Cronbach's alpha) was checked for each frequency subscale.

Preliminary analysis of variables was conducted to test for normality and suitability for parametric testing. Associations between frequencies of use of the five sensory approaches (overall use of sensory interventions, weighted modalities, sensory room, sensory groups, and sensory assessments) and other variables were considered in two‐ways. First, analyses of variance (ANOVAs) were conducted to consider associations between the five sensory approaches and categorical demographic variables (unit type, discipline, gender). Second, Pearson's correlation analyses were conducted with continuous variables (amount of training, age, years of experience in mental health practice) and the five sensory approaches. A significance level of *P* < 0.05 was adopted.

Qualitative data from free‐text questions were analysed using summative content analysis (Hsieh & Shannon, 2005) to summarise the different types of sensory approaches used by participants. Content analysis started with familiarisation with the data, following which key words/concepts were identified and quantified (Hsieh & Shannon, 2005) with the purpose of establishing categories of sensory approaches used by participants. Categories were initially developed by primary author (LW) and checked independently by co‐authors (PM & SB) to enhance rigour of the final category structure.

## 3 RESULTS

### 3.1 Participant characteristics

A total of 211 questionnaires were received (response rate of approximately 50% of eligible participants in the service studied), of which 183 were analysed. Three questionnaires were excluded due to large amounts of missing data, and an additional 25 questionnaires were excluded as participants did not work directly with consumers and therefore did not answer questions on frequency of use of sensory approaches. Participant characteristics are summarised in Table 1.

### 3.2 Proportion of consumers with whom they used sensory interventions

Of the participants who responded to the question regarding the proportion of consumers with whom they used sensory interventions (*n* = 163), most (*n* = 153, 89%) indicated they used sensory interventions with consumers to some extent (see Table 1). However, over half of these participants used sensory interventions with ≤25% of consumers.

### 3.3 Open question: Types of sensory interventions used

Participants who used sensory interventions were invited to list the specific types of interventions they used. Ninety‐seven participants (i.e., 53% of total participants) responded, each listing between 2 and 14 different types of sensory interventions. Content analysis was used to categorise participants' responses into different types of sensory approaches (see Table 2).

**TABLE 2:** Summary of types of sensory approaches used, *n =* 97

	Sensory approaches used	Response frequency^a^ *n* (%)
Sensory interventions	Sensory room	21 (22)
	Weighted modalities (*weighted blankets, vests, hoodies, toys*)	29 (29)
	Sensory reduction (*e.g., use of quiet spaces, low stimulus spaces, ear plugs and/or soft lightning*)	11 (11)
	Sensory kit/sensory cupboard/‘Calm Down’ box use on ward^b^	9 (9)
	Sensory groups	8 (8)
	Sensory Diet, Sensory Plan, Sensory Assessment, Personal Safety Plan	7 (7)
	Sensory exploration with discussion & education	5 (5)
Sensory activities stimulating different senses	Smell: aromatherapy (*e.g., use of essential oils, scented sprays, diffusers*)	47 (48)
	Tactile: fidget items (*e.g. stress balls, textured, tactile items, putty, squishy toys*)	45 (46)
	Auditory: music (*e.g., iPads music Apps, relaxing music, rain sounds*)	43 (44)
	Taste (*e.g., sour lollies, herbal teas*)	24 (25)
	Temperature change (*e.g., heat or cold packs, warm or cold drinks, encourage warm showers*)	12 (12)
	Visual (*e.g., iPad Apps, photos, items that light, playing relaxing scenery on DVD*)	12 (12)
	Vestibular (*e.g., rocking chair*)	2 (2)
	Oral motor (*e.g., blowing bubbles*)	2 (2)
Sensory‐based activities	Art/craft/games/puzzles (*e.g., mindful colouring, clay modelling, card games*)	21 (22)
	Relaxation techniques (*e.g., meditation, deep breathing techniques*)	21 (22)
	Physical activity: low intensity (*e.g., isometric, tai chi, walking*)	7 (7)
	Physical activity: high intensity (*e.g., playing ball*)	6 (6)
	Mindfulness techniques	6 (6)
	Touch/massage (*e.g., massage chair, massage roller, pressure balls*)	6 (6)
	Other (*e.g., recreational room, encourage sensory occupations*)	6 (6)
	Grounding techniques (*e.g., standing in the dirt*)	2 (2)

^a^ The response frequency is calculated from the 97 participants who responded to this question (i.e., 53% of the total number of participants).

^b^ Sensory kits/sensory cupboard/’Calm Down’ box contain a variety of sensory items that include but are not restricted to herbal teas, stress balls, fidget items, mindful colouring, fragrant body/hand cream, and sour or peppermint lollies.

### 3.4 Frequency and reason for use of sensory interventions

The third research question asked how frequently, and for what purpose, different sensory interventions are used. Participants reported *how frequently* they used sensory interventions *overall*, as well as the frequency of specific sensory interventions, including weighted modalities, sensory rooms, sensory assessments/plans, and sensory groups. As seen in Table 3, less than 40% of participants reported frequently using sensory interventions *overall*. The least‐used sensory interventions were sensory assessments and sensory groups.

**TABLE 3:** Frequency of use and reasons for use of different sensory approaches, *n* = 183

Subscale	Questions	Valid responses *n*	Never *n* (%)	Rarely–sometimes *n* (%)	Frequently–very frequently *n* (%)	Mean (SD)	Cronbach's alpha
Frequency of use of sensory interventions overall	I facilitate consumers to *use* sensory interventions and or sensory equipment	182	19 (10.4%)	94 (51.6%)	69 (37.9%)	2.27 (.64)	0.90
	I use sensory interventions with consumers to *reduce their anxiety* and *agitation*	181	17 (9.4%)	88 (48.6%)	76 (42.0%)	2.32 (.64)	
	I use sensory interventions with consumers to *assist* with *regulating their emotions*	182	17 (9.3%)	98 (53.8%)	67 (36.8%)	2.27 (.62)	
	I use sensory modulation approaches with consumers to *prevent the use* of *seclusion* and *restraint*. (*unavailable: 12, 6.6%*)^a^	181	45 (26.6%)	76 (45.0%)	48 (28.4%)	1.91 (.80)	
	I *educate* consumers about sensory interventions that can be used to assist in their recovery	180	26 (14.4%)	79 (43.9%)	75 (41.7%)	2.19 (.77)	
Frequency of use of comfort or sensory room	I *facilitate* consumers to use the ‘*comfort room*’ or sensory room (*unavailable: 27; 15%*)^a^	180	33 (41.7%)	55 (35.9%)	65 (42.5%)	2.20 (.77)	
Frequency of use of weighted modalities	I *facilitate* consumers to use *weighted modalities* (weighted blankets, toys or vests) (*unavailable: 10, 5.5%*)^a^	181	39 (22.7%)	102 (59.3%)	31 (18.0%)	1.95 (.63)	0.93
	I use *weighted modalities* with consumers to reduce their *distress* (*unavailable: 12; 6.6%*)^a^	181	49 (29.0%)	86 (50.9%)	34 (20.1%)	1.91 (.69)	
	I use weighted modalities to assist consumers improve their *sleep* (*unavailable: 12; 6.6%*)^a^	181	57 (33.5%)	91 (53.3%)	22 (12.9%)	1.79 (.77)	
Frequency of use of sensory assessment and plans	I complete *personal safety plan* ^b^ with consumers (*unavailable: 2; 1.1%*)^a^	180	55 (30.6%)	84 (46.7%)	41 (22.8%)	1.92 (.72)	0.86
	I *refer* consumers to have a Sensory Profile^c^ completed (*unavailable: 2; 1.1%*)^a^	180	64 (36.0%)	77 (43.3%)	37 (20.8%)	1.84 (.73)	
	I complete *Sensory Profiles* with consumers (*unavailable: 3; 1.6%*)^a^	180	99 (55.9%)	57 (32.2%)	21 (11.9%)	1.55 (.69)	
	I complete *sensory plans* ^d^ with consumers (*unavailable: 3; 1.6%*)^a^	180	101 (57.1%)	55 (31.1%)	21 (10.1%)	1.54 (.69)	
	I complete *sensory* checklist^e^ with consumers (*unavailable: 2; 1.1%*)^a^	180	90 (50.6%)	72 (40.2%)	16 (9.0%)	1.5 (.65)	
Frequency use of sensory groups	I *facilitate* sensory *groups* with consumers (*unavailable: 6; 3.2%*)^a^	182	90 (51.1%)	61 (34.7%)	25 (14.2%)	1.63 (.72)	

^a^ Participants could indicate if the sensory intervention or equipment was unavailable in their work unit.

^b^ Personal safety plans identify triggers, warning signs, and individualised sensory strategies (Chalmers et al., 2012).

^c^ The Adolescent/Adult Sensory Profile is a validated assessment tool that evaluates an individual's patterns of sensory processing (Brown & Dunn, 2002).

^d^ Sensory plans summarise individual warning signs, triggers, and helpful and unhelpful sensory strategies to use in times of distress.

^e^ Sensory checklists help identify activities that can assist in reducing or preventing distress (Champagne, 2011).

Participants also reported the frequency they used sensory interventions *overall* and weighted modalities (if available) for *specific reasons*. Results are presented in Table 2. The most frequent reason for using sensory interventions *overall* was to reduce consumer anxiety and agitation, followed by improving a consumer's emotional regulation. Most participants *infrequently* used sensory interventions *overall* to reduce the use of seclusion and restraint. Participants indicated they used weighted modalities *infrequently* to *(1) reduce distress* and *(2) improve sleep* with consumers.

### 3.5 Factors influencing frequency of use

Statistical analyses were performed to answer the fourth research question: whether there was an association between frequencies of use of different sensory approaches and *demographic/clinical factors* (e.g., unit type, discipline, amount of training, years of experience in mental health, and gender). The different sensory approaches used in correlational analysis were (1) sensory interventions *overall* subscale, (2) weighted modalities subscale, (3) sensory room item, (4) sensory group item, and (5) sensory assessment subscale. Confirmatory factor analysis performed on frequency subscales confirmed the relationship between items in each subscale: (1) Frequency of use of sensory interventions *overall* (*χ* ^2^ = 0.88; *df* = 10; *P* ≤ 0.001); (2) Frequency of use of weighted modalities (*χ* ^2^ = 0.75; *df* = 3; *P* ≤ 0.001); and (3) Frequency of use of sensory assessments (*χ* ^2^ = 0.75; *df* = 3; *P* ≤ 0.001).

As evident in Table 4, the factor most strongly associated with frequency of all types of sensory approaches was the amount of training participants had attended. There was a significant positive correlation between more frequent use of sensory approaches (sensory interventions *overall*, weighted modalities, sensory room, sensory groups, and sensory assessments) and having attended more training sessions. Discipline (occupational therapy) was also associated with higher frequency of use for two of the sensory approaches (weighted modalities and sensory assessments). Occupational therapists and occupational therapy assistants were more likely to use sensory interventions *overall*. The unit type (acute inpatient unit) was also associated with more frequent of use of sensory rooms, weighted modalities, and sensory groups while this was not evident for secure mental health units or residential community care units. Participants working in acute inpatient units had not attended more training than participants working in other units. There were no associations between frequencies of use and either gender or participants' years of experience in mental health. Practitioner age was significantly negatively correlated only with weighted modalities, with frequency of use decreasing with increased age.

**TABLE 4:** Comparison between frequency of use of different sensory interventions and occupational/demographic factors

	Sensory interventions overall *n* = 171	Weighted modalities *n* = 144	Sensory rooms *n* = 166	Sensory groups *n* = 163	Sensory assessments *n* = 163
Dependent variable	ANOVA statistic				
Unit type	$f\left(5,174\right)=2.14$	$f\left(3,170\right)=3.51$ *	$f\left(5,147\right)=4.69$ ***	$f\left(5,170\right)=2.51$ *	$f\left(5,166\right)=1.66$
Discipline	*f* (7,172) = 5.79***	$f\left(7,173\right)=2.34$ *	$f\left(7,145\right)=1.90$	$f\left(7,168\right)=10.01$ ***	$f\left(7,164\right)=5.79$ ***
Gender	$f\left(1,169\right)=2.69$	$f\left(1,169\right)=2.45$	$f\left(1,142\right)=3.71$	$f\left(2,,164\right)=1.10$	$f\;\left(2,164\right)=1.62$
Dependent variable	Pearson's correlation coefficient between dependent variable and frequency of use				
Amount of training	*r* = 0.43**	*r* = 0.30**	*r* = 0.32**	*r* = 0.47**	*r* = 0.43**
Age	*r* = −0.06	*r* = −0.20**	*r* = −0.15	*r* = −0.06	*r* = −0.10
Years in mental health	*r* = 0.01	*r* = −0.04	*r* = −0.05	*r* = −0.11	*r* = −0.61

Abbreviation: ANOVA, analysis of variance.

* *P* ≤ 0.05.

** *P* ≤ 0.01.

*** *P* ≤ 0.001.

## 4 DISCUSSION

This study investigated the current pattern of use of sensory approaches across psychiatric units in one metropolitan mental health service in Australia. It explored the extent of their use, types of sensory approaches used, frequency and purpose of use, and demographic and clinical factors associated with frequency of use. Prior to this study, little was known about how mental health clinicians use sensory approaches in every day clinical practice. Despite government reports and research highlighting problems sustaining implementation of sensory approaches in some services in Australia (Wright et al., 2017, 2020), available documents did not reveal the pattern of use of sensory approaches or the demographic or clinical factors that influenced frequency of use. This information is important to inform the development of implementation strategies to improve the use and sustainability of sensory approaches in psychiatric units.

### 4.1 Proportion of consumers receiving sensory interventions

Half of the participants indicated that they used sensory interventions either not at all or with less than a quarter of consumers with whom they worked. Importantly, this finding does not reveal the number of consumers needing or receiving sensory interventions, which may also have impacted on results. Nevertheless, this limited use of sensory interventions is surprising considering government recommendations, and there is growing neuro‐scientific evidence that people with mental illness experience sensory processing difficulties, which impact their functioning (Bailliard & Whigham, 2017). Although reasons for non‐use in this study are unclear, findings are supported by previous research investigating barriers to the use of sensory approaches in psychiatric units. Qualitative research has shown that mental health clinicians may not use sensory approaches if they believe it is not within their role, perceive a lack of time or support from colleagues to its use, or lack skills or confidence for its use (Wright et al., 2020). Lack of knowledge and awareness about how to use sensory approaches in mental health care has also been linked to reduced use of sensory interventions (Martin & Suane, 2012; Wright et al., 2021). In addition, mental health clinician's concerns about potential risks of sensory equipment and rooms have been identified as a factor that may influence the decision to use sensory approaches with consumers (Machingura et al., 2021; Wiglesworth & Farnworth, 2016; Wright et al., 2020). Regardless, the limited use by many participants contrasts with the growing evidence that supports the use of sensory approaches in mental health care. Investigating these challenges in larger samples may help determine the extent to which each issue may influence the use of sensory approaches.

#### 4.1.1 Types of sensory approaches

Although use of sensory approaches was less than ideal, participants who did use these approaches reported using a full range of sensory experiences, including aromatherapy, music, fidget items, weighted modalities, and art. This variety could reflect the focus on individual sensory preferences that supports person‐centred and trauma‐informed care (Craswell et al., 2020). The use of individualised sensory approaches is consistent with the work of Champagne (2011) who inspired the introduction of these approaches into mental health care.

#### 4.1.2 Frequency of use

This study investigated mental health clinicians' self‐reported frequency of their use of *sensory interventions overall*, as well as of different types of sensory interventions. How often sensory interventions were used varied depending on the type of sensory intervention, with sensory assessments and sensory groups least used. Where available, sensory rooms were used most frequently. Frequency of use of specific sensory interventions has been previously investigated, for example, for sensory rooms (Chalmers et al., 2012; Novak et al., 2012; Sivak, 2012) and personal safety plans (Lee et al., 2010). However, no study has compared the frequency of use for a range of *different* sensory interventions. Studies that have considered multiple sensory interventions (including sensory rooms, personal safety plans, and sensory groups) (Espinosa et al., 2015; Yakov et al., 2017) have focussed on measuring clinical outcomes rather than frequency of use. These studies have reported pre‐post comparison of clinical outcomes such as rates of seclusion and restraint or incidents of aggression, with no comment on actual use by health professionals or consumers. While recommendations in government documents indicate that they should be available for use by all consumers admitted to mental health units, they do not specify the frequency with which sensory interventions should be used.

Of those participants who had access to sensory rooms, less than half facilitated the use of the sensory room with consumers *frequently to very frequently*. National guidelines (Australian Health Ministers' Advisory Council, 2016) suggest having sensory approaches, including spaces and sensory options, available in inpatient units, and the therapeutic benefits of sensory rooms are now well established (Cheng et al., 2017; Novak et al., 2012). Barriers to use of sensory rooms include lack of training (e.g., Martin and Suane (2012) and poor maintenance of the rooms (Wright et al., 2020). Addressing the lack of training for 34.4% of participants in the present study who had never received training in sensory approaches may contribute to increased use of sensory rooms. In addition, maintenance of sensory rooms may improve their use; however, as the condition of the sensory rooms across multiple sites within the large health service district in the present study was unknown, it is not possible to comment on this as a possible reason for lack of use.

In addition to having sensory spaces available in psychiatric units, Australian government and state mental health guidelines/policies/standards recommend that personal safety plans (or personal prevention plans) be completed with consumers admitted to psychiatric units (Australian Health Ministers' Advisory Council, 2016; New South Wales Government, 2020; South Australian Health, 2017; Tasmanian Health, 2013; Victorian Health, 2017; Western Australian Health, 2015). Nevertheless, only approximately 20% of the participants in our study routinely used personal safety plans with consumers in their clinical practice. Consistent with this finding, Chalmers et al. (2012) also reported problems with the plan being used consistently, although highlighted the usefulness of personal safety plans to identify sensory strategies to manage consumer distress. They identified barriers to completing the personal safety plans as a lack of time and amount of paperwork that staff need to complete. The barriers to completing personal safety plans in the health service studied warrant further consideration to inform implementation strategies to address this issue.

### 4.2 Reasons for use

The most frequent reasons participants identified for using sensory approaches with consumers was to reduce anxiety and agitation and assist with regulating their emotions. This is consistent with evidence indicating the efficacy of sensory approaches to reduce distress and agitation and assist with emotional regulation (Chalmers et al., 2012; Cheng et al., 2017). Similarly, the National consensus statement on recognising and responding to deteriorating mental state in health‐care settings recommends the use of sensory approaches as an intervention to improve a person's emotional state (Australian Commission on Safety and Quality in Health Care, 2017). Interestingly, while government recommendations suggest using sensory approaches to prevent the use of seclusion and restraint, over a quarter of our participants reported that they *never* use sensory approaches for this purpose. As research increasingly supports the usefulness of sensory approaches to reduce seclusion and restraint (Cummings et al., 2010; Lloyd et al., 2014), it is unclear why most participants do not frequently use sensory approaches to reduce the use of seclusion and restraint. Some barriers to general use of sensory approaches have been discussed. Additionally, participants may lack the belief that sensory approaches are effective to reduce seclusion and restraint or be unsure at what point sensory approaches should be used to optimise outcomes and minimise risk. Wright et al. (2020) found that some mental health clinicians believed sensory interventions were too risky, particularly for use with highly distressed consumers or those in psychiatric intensive care units where PRN medication were preferred. Ideally, sensory interventions should be used as an early intervention strategy to prevent distress and agitation thereby reducing the need for seclusion and restraint. Another consideration is the lack of availability of sensory tools and equipment in the psychiatric units; some participants in our study indicated they did not have sensory rooms (15%) or weighted modalities (5.5%) in their workplace. The lack of sensory equipment and resources has been previously identified to limit the use of sensory approaches (Machingura et al., 2021; Wright et al., 2020).

### 4.3 Demographic and occupational factors influencing use

The final aim of this study was to consider associations between frequency of use of sensory approaches and demographic/occupational characteristics. As noted earlier, a relationship between frequency of use and the amount of *training* in sensory approaches attended was identified for all sensory approaches. While national guidelines recommend that mental health staff should be trained in sensory approaches, the amount and type of training are not specified (Australian Health Ministers' Advisory Council, 2016). Most participants in this study had attended at least one type of training, suggesting that more training is needed to improve patterns of use. Consistent with this, studies investigating the use of sensory approaches in inpatient psychiatric units consistently recommend, including initial and ongoing training in sensory processing and sensory approaches (Azuela & Robertson, 2016; Chalmers et al., 2012; Sutton & Nicholson, 2011; Wright et al., 2020). More training options (e.g., beginner, intermediate, and advanced levels of training), and provision of refresher training in sensory approaches, as part of an implementation strategy, may improve use of sensory approaches. As discussed earlier, other factors, such as availability of sensory materials and resources, also need to be addressed, to improve use of sensory approaches in clinical practice.

Clinician *age* was associated with greater use of sensory approaches in the present study, with younger mental health clinicians more likely to use weighted modalities. This is the first study to reveal this finding, and reasons remain unclear, including reasons for why this effect was noticed only for weighted modalities. It was considered that younger participants may have attended more training in sensory approaches than other participants, but this was not found. The increased use of weighted modalities by younger participants may be related to their belief that they are effective to reduce distress and improve sleep.

In previous studies, sensory approaches have been shown to be used more frequently with female consumers in psychiatric units (Novak et al., 2012; Sivak, 2012). Interestingly, *gender* of participants was not associated with frequency of use in this study. Finally, the *discipline* of occupational therapy was associated with increased frequency of use for all sensory approaches except for sensory rooms. This is not surprising as occupational therapists are frequently identified as the professional lead in the implementation of sensory approaches in mental health services (Scanlan & Novak, 2015). Mental health services wanting to increase the use of sensory approaches in inpatient units should consider the importance of having access to occupational therapists.

## 5 LIMITATIONS

Limitations in this study should be acknowledged. Participants worked in one health district in Queensland; therefore, results may not represent all peer workers and mental health clinicians working in psychiatric units. Even though 50% of staff working across the mental health inpatient units answered the questionnaire, participant bias could factor in the results as selection of participants was not randomised. For example, only staff interested in using sensory approaches may have answered the questionnaire. In addition, use of self‐report questions contributes bias, with participants providing their perceptions of their use of sensory approaches and not an objective measure of their actual use. These biases may have produced results that over‐ or underestimated the frequency of use of sensory approaches. Another limitation is that participants were not asked how consumer preference/choice influenced their use of different sensory approaches, which may have influenced results. Although the questionnaire used was supported by confirmatory factor analysis and revealed good internal consistency, it was not standardised; thus, further research is required to consider its reliability and validity. Finally, findings about the reasons participants used sensory approaches with consumers were limited to a fixed number and did not provide an option to describe other reasons sensory approaches were used. Further research is needed to address these limitations.

## 6 CONCLUSION

While sensory approaches have been recommended in mental health inpatient care for over 15 years, little is known about their pattern of use and demographic/occupational factors influencing use by mental health clinicians working in psychiatric units. This study is the first to explore the pattern and frequency of use of sensory approaches by mental health clinicians in their routine clinical practice in psychiatric units. Findings provide insight into how mental health clinicians use sensory approaches and the demographic/occupational factors that influence their frequency of use. Findings highlight a gap between recommendations to use sensory approaches in psychiatric inpatient care and their actual use. Training in sensory approaches, including refresher training, and access to occupational therapists should be considered by mental health services experiencing challenges implementing and sustaining the use of sensory approaches. Further research is required to establish why sensory approaches were only used with a limited proportion of consumers and why some approaches, such as personal safety plans, were used infrequently, considering recommendations for their use in psychiatric units.

## CONFLICT OF INTEREST

SB & PM declare no competing interests. LW is employed at the Metro North Mental Health and facilitated sensory modulation training across the service.

## AUTHOR CONTRIBUTIONS

All authors were involved in the design and planning of this study. LW was responsible for the dissemination of the survey and analysis of the data. PM and SB provided guidance on the data analysis. All authors participated in the drafting of the manuscripts and approved the final manuscript.

## Data Availability

Data available on request due to privacy/ethical restrictions.
